# PARP Inhibitor Decreases Akt Phosphorylation and Induces Centrosome Amplification and Chromosomal Aneuploidy in CHO-K1 Cells

**DOI:** 10.3390/ijms23073484

**Published:** 2022-03-23

**Authors:** Masakazu Tanaka, Masatoshi Mushiake, Jun Takahashi, Yuka Sasaki, Sachiko Yamashita, Chieri Ida, Mitsuko Masutani, Masanao Miwa

**Affiliations:** 1 Department of Microbiology, Kansai Medical University, Osaka 573-1010, Japan; 2 Division of Neuroimmunology, Joint Research Center for Human Retrovirus Infection, Kagoshima University, Kagoshima 890-8544, Japan; 3 Faculty of Bioscience, Nagahama Institute of Bio-Science and Technology, Nagahama 526-0829, Japan; kid610809@gmail.com (M.M.); roubai1988@yahoo.co.jp (J.T.); yamatico35@gmail.com (S.Y.); m_miwa@nagahama-i-bio.ac.jp (M.M.); 4 Laboratory of Collaborative Research, Division of Cellular Signaling and Radioisotope Division, National Cancer Center Research Institute, Tokyo 104-0045, Japan; sasaki-y@cc.osaka-dent.ac.jp (Y.S.); mmasutan@nagasaki-u.ac.jp (M.M.); 5 Department of Molecular and Genomic Biomedicine, CBMM, Nagasaki University Graduate School of Biomedical Sciences, Nagasaki 852-8523, Japan; 6 Department of Applied Life Sciences, College of Nagoya Women’s University, Nagoya-shi 467-8610, Japan; chieri0503@gmail.com

**Keywords:** Akt, cell proliferation, centrosome amplification, chromosome aneuploidy, PARP inhibitors, polyADP-ribosylation

## Abstract

Cancer cells are known to have chromosomal number abnormalities (aneuploidy), a hallmark of malignant tumors. Cancer cells also have an increased number of centrosomes (centrosome amplification). Paradoxically, cancer therapies, including γ-irradiation and some anticancer drugs, are carcinogenic and can induce centrosome amplification and chromosomal aneuploidy. Thus, the processes of carcinogenesis and killing cancer cells might have some mechanisms in common. Previously, we found that the inhibitors of polyADP-ribosylation, a post-translational modification of proteins, caused centrosome amplification. However, the mechanism of action of the inhibitors of polyADP-ribosylation is not fully understood. In this study, we found that an inhibitor of polyADP-ribosylation, 3-aminobenzamide, caused centrosome amplification, as well as aneuploidy of chromosomes in CHO-K1 cells. Moreover, inhibitors of polyADP-ribosylation inhibited AKT phosphorylation, and inhibitors of AKT phosphorylation inhibited polyADP-ribosylation, suggesting the involvement of polyADP-ribosylation in the PI3K/Akt/mTOR signaling pathway for controlling cell proliferation. Our data suggest a possibility for developing drugs that induce centrosome amplification and aneuploidy for therapeutic applications to clinical cancer.

## 1. Introduction

It is well known that cancer cells have genetic instability, including many mutations, which drives unlimited cell proliferation [1]. At the same time, cancer cells have centrosome amplification and chromosomal number abnormalities (aneuploidy) [2,3]. Although agents such as γ-irradiation and the anti-neoplastic chemicals are used for cancer therapy, some of them are carcinogenic [4] and can induce mutations, centrosome amplification and chromosomal aneuploidy [5,6]. These paradoxical phenomena suggest that genomic instability causes both cell transformation as well as cancer cell death. The above findings provide a possibility to develop drugs causing centrosome amplification and aneuploidy as another strategy to treat cancer. Thus, it is important to understand the relationship between centrosome amplification, aneuploidy and tumor formation. In 1914, from his work with sea urchins, Boveri speculated that malignant tumors could be caused by unbalanced combinations of chromosomes through asymmetrical mitosis, which was caused by multiple divisions of the centrosome or by unparalleled division of centrosomes [7,8].

It is well known that post-translational modifications, such as phosphorylation, are important as a molecular mechanism of cell transformation [1]. PolyADP-ribosylation (PARylation) is a post-translational modification with a long chain of poly(ADP-ribose) which modifies various proteins and regulates various cellular functions [9,10]. One of the functions of PARylation is involvement in DNA strand break repair, and the inhibitors of poly(ADP-ribose) synthesis have recently demonstrated clinical synthetic lethality to ovarian and breast cancers with homologous recombination repair deficiency [11,12,13,14].

During our work on PARylation, we found that the inhibitors of PARylation or poly(ADP-ribose) polymerase 1 (PARP1)-knockout cells exhibited centrosome amplification [15]. In this study, we show that the PARP inhibitor could cause both centrosome amplification and aneuploidy and also inhibit the signal transduction pathway of Akt, which could explain one of the mechanisms of inhibiting cell proliferation. These findings might be taken into consideration for developing drugs to target DNA.

## 2. Results

### 2.1. PARP Inhibitors Induced Centrosome Amplification in CHO-K1 Cells

**Figure 1:**
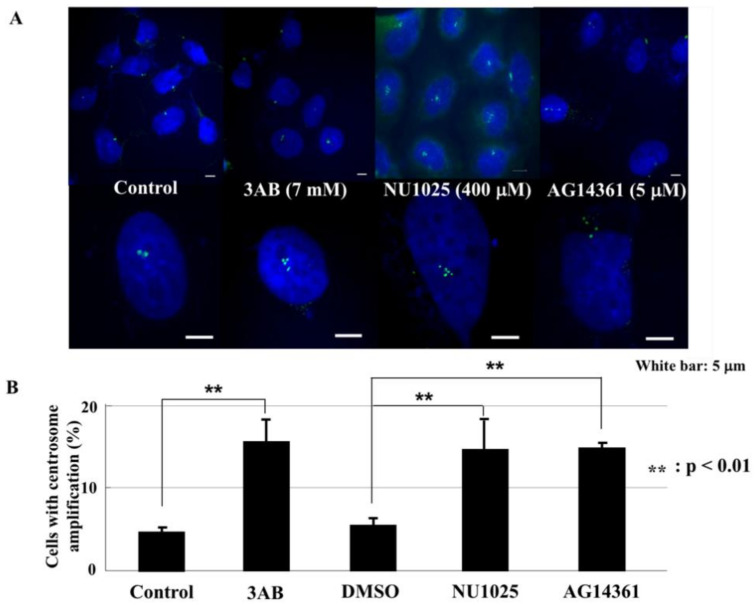
PARP inhibitors induced centrosome amplification in CHO-K1 cells. (**A**) CHO-K1 cells were cultured for 72 h with PARP inhibitors and stained with anti-γ-tubulin antibody. (**B**) Cells with centrosome amplification by PARP inhibitors were summarized. Significance was assessed against the control. ** *p* < 0.01. PARP inhibitor 3AB was dissolved directly in the culture medium, and NU1025 and AG14361 were first dissolved in DMSO and diluted by the culture medium to the designated concentration. The final concentration of DMSO was 0.01%, which was not cytotoxic.

We have previously shown that PARP inhibitors can cause centrosome amplification in CHO-K1 cells [16]; therefore, we defined the concentrations of three PARP inhibitors that cause centrosome amplification to similar percentages of CHO-K1 cells after culturing for 72 h. 3-Aminobenzamide (3AB), NU1025 and AG14361 induced abnormalities in the number of centromeres in about 17% of CHO-K1 cells at 7 mM, 400 μM and 5 μM, respectively (Figure 1A,B).

### 2.2. PARP Inhibitors Inhibited Cell Proliferation without Changes in Flow Cytometric Pattern in CHO-K1 Cells

**Figure 2:**
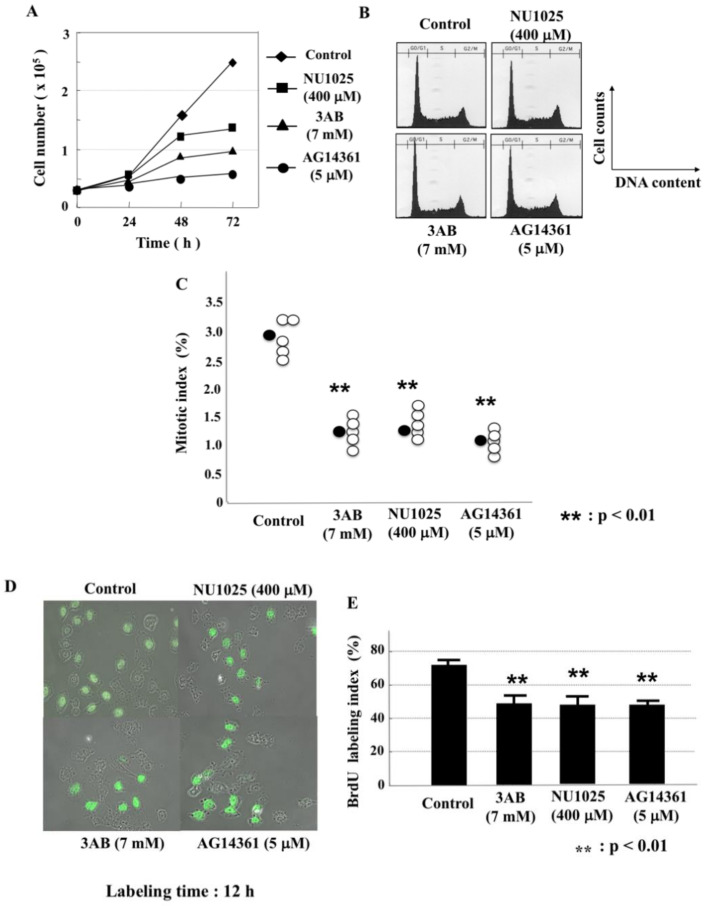
The suppression of cell proliferation by PARP inhibitors without changes in flow cytometric patterns. CHO-K1 cells were cultured in the presence of various PARP inhibitors for 72 h. (**A**) The viable cells were counted with trypan blue dye exclusion. (**B**) Flow cytometric pattern after 48 h with or without PARP inhibitors. (**C**) The mitotic index was calculated after culturing for 72 h with colcemid for 6 h. *n* = 5. White circles show the percentage of mitosis in each experiment, and black circles show the mean values of 5 experiments. Significance was assessed against the control with two-tailed Student’s *t*-tests. ** *p* < 0.01. (**D**) Fluorescence microscopic views of the incorporation of BrdU after culturing for 12 h in 10 μM BrdU for 12 h. (**E**) Cells which incorporated BrdU were counted. Significance was assessed against the control. ** *p* < 0.01.

When CHO-K1 cells were cultured in the presence of PARP inhibitors—3AB, NU1025 and AG14361—the cell proliferation was inhibited (Figure 2A) without significant changes in flow cytometric patterns (Figure 2B). The mitotic index and 5-bromodeoxyuridine (BrdU) incorporation were also significantly inhibited (Figure 2C–E), suggesting that progression of the whole cell cycle was inhibited.

### 2.3. PARP Inhibitor Induced Aneuploidy in CHO-K1 Cells

**Figure 3:**
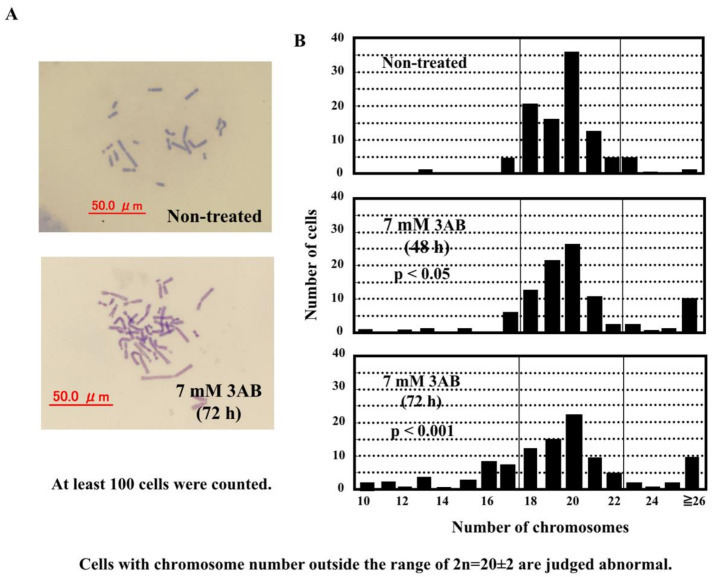
3AB, a PARP inhibitor, induced aneuploidy of chromosomes. (**A**) Photograph of metaphase of cells treated with or without 7 mM 3AB for 72 h. (**B**) Histogram of cells (%) with a defined number of chromosomes. At least 100 metaphases were analyzed. The number of cells with chromosome number outside of the range of 20 ± 2 was judged as abnormal. Significance was analyzed with Fisher’s exact test.

When CHO-K1 cells were incubated with 3AB, which is neither mutagenic nor toxic to CHO-K1 cells at concentrations of 7 mM [16,17,18], aneuploidy of chromosomes was significantly observed (Figure 3A,B). To the best of our knowledge, this is the first observation of the induction of aneuploidy of chromosomes induced by a non-toxic PARP inhibitor in cultured cells.

### 2.4. PARP Inhibitors Inhibited the Phosphorylation of Akt in CHO-K1 Cells

**Figure 4:**
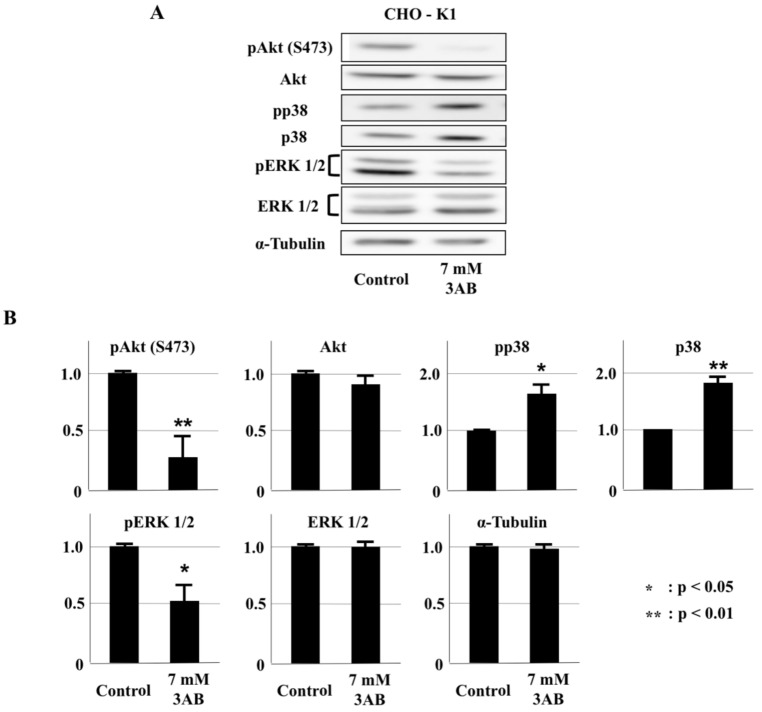
The PARP inhibitor 3AB reduced phosphorylated Akt levels. (**A**) The cell proliferation signaling proteins p38 MAPK, ERK 1/2 and Akt, together with their phosphorylated proteins, were detected by immunoblot analysis after CHO-K1 cells were treated with 7 mM 3AB for 48 h. α-Tubulin is shown as a loading control. (**B**) The relative intensities of Western blots from 3 experiments are summarized. Significance was assessed against the control. * *p* < 0.05. ** *p* < 0.01.

PARP inhibitors inhibited cell proliferation at non-toxic concentrations; therefore, we thought there was some disturbance in certain signaling pathways. In this study, we first examined the pathways known to be involved in cell proliferation and cell survival (AKT, p38MAPK, ERK1/2) using PARP inhibitors. We found that the phosphorylation of Akt, detected by the antibody to recognize the phosphorylation of human AKT at S473, was significantly inhibited (Figure 4A,B, Supplemental Information Figure S1).

### 2.5. Akt inhibitors Inhibited polyADP-Ribosylation In Vivo, and Induced Centrosome Amplification and Aneuploidy in CHO-K1 Cells

**Figure 5:**
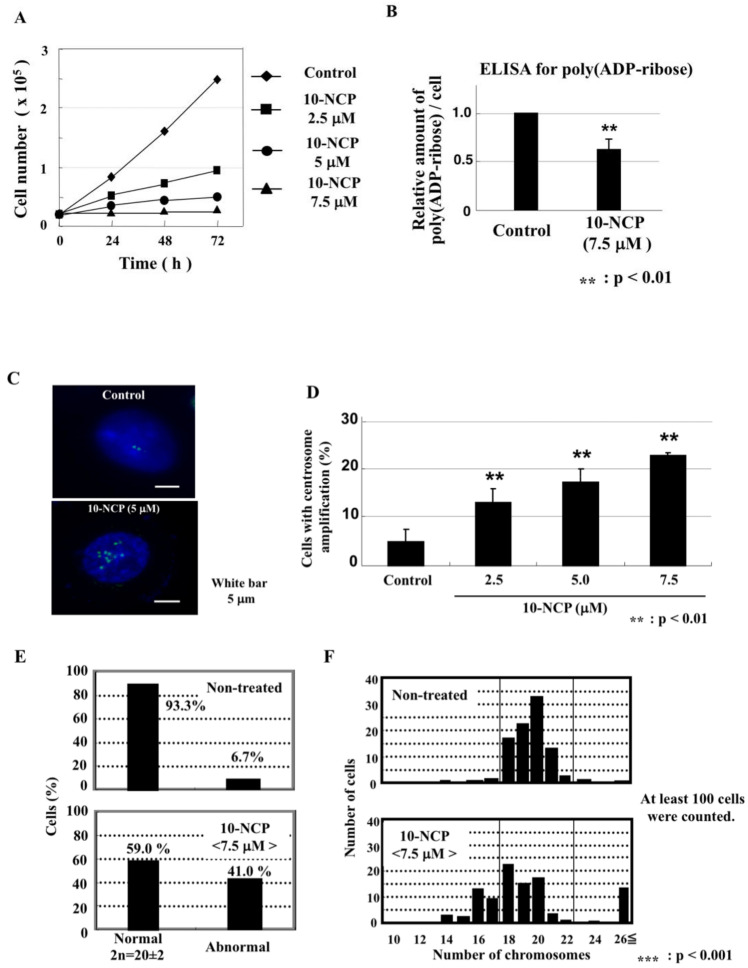
An Akt inhibitor, 10-NCP, inhibited in vivo polyADP-ribosylation and induced centrosome amplification and aneuploidy of chromosomes. (**A**) Inhibition of cell proliferation with indicated concentrations of 10-NCP. (**B**) Relative in vivo level of poly(ADP-ribose) per cell after culturing cells with 10-NCP for 48 h, determined by ELISA. (**C**) Cells were cultured for 48 h with or without 5 μM 10-NCP and immunostained with an antibody against γ-tubulin. (**D**) Percentage of cells with centrosome amplification. Significance was assessed against the control. ** *p* < 0.01. (**E**) Cells with the number of chromosomes outside of the range of 20 ± 2 were defined as abnormal. Significance was analyzed with Fisher’s exact test. (**F**) Histogram of cells with a defined number of chromosomes. At least 100 metaphases were analyzed. Significance was analyzed with Fisher’s exact test against the control. *** *p* < 0.001.

As expected, an AKT inhibitor, 10-NCP, suppressed the pAkt levels of CHO-K1 cells dose-dependently (data not shown) and reduced cell proliferation (Figure 5A). PARP inhibitors decreased cell proliferation and decreased pAkt levels in CHO-K1 cells; therefore, we hypothesized that the AKT inhibitor could decrease in vivo levels of poly(ADP-ribose), the marker of PARylation. Indeed, 10-NCP significantly reduced the in vivo level of poly(ADP-ribose) (Figure 5B).

The AKT inhibitor inhibits in vivo levels of poly(ADP-ribose); thus, it might also induce centrosome amplification and chromosomal aneuploidy. Actually, we found that 10-NCP induced centrosome amplification (Figure 5C,D) in a concentration-dependent manner and also caused aneuploidy of chromosomes (Figure 5E,F).

**Figure 6:**
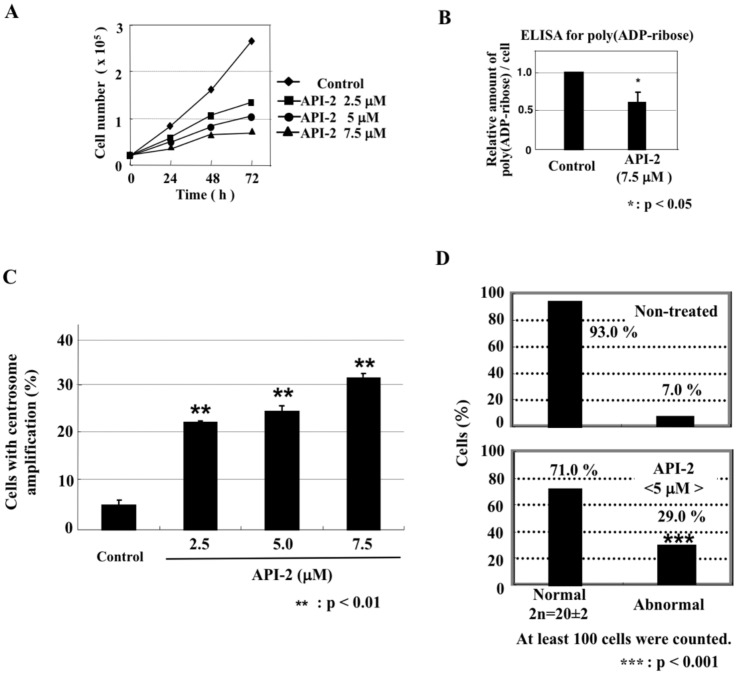
Another Akt inhibitor, API-2, also induced centrosome amplification and aneuploidy of chromosomes. (**A**) Inhibition of cell proliferation with indicated concentrations of API-2. (**B**) Relative in vivo level of poly(ADP-ribose) per cell, determined by ELISA, after culturing cells with API-2 for 48 h. (**C**) Percentage of cells with centrosome amplification. Significance was assessed against the control. ** *p* < 0.01. (**D**) Cells with the number of chromosomes outside of the range of 20 ± 2 were defined as abnormal. Significance was analyzed with Fisher’s exact test against the control. *** *p* < 0.001.

Another AKT inhibitor, API-2, also dose-dependently inhibited cell proliferation (Figure 6A), reduced the in vivo level of poly(ADP-ribose) (Figure 6B) and induced centrosome amplification (Figure 6C) and aneuploidy of chromosomes (Figure 6D).

### 2.6. AKT Inhibitors Did Not Inhibit PARP Enzyme Activity

**Figure 7:**
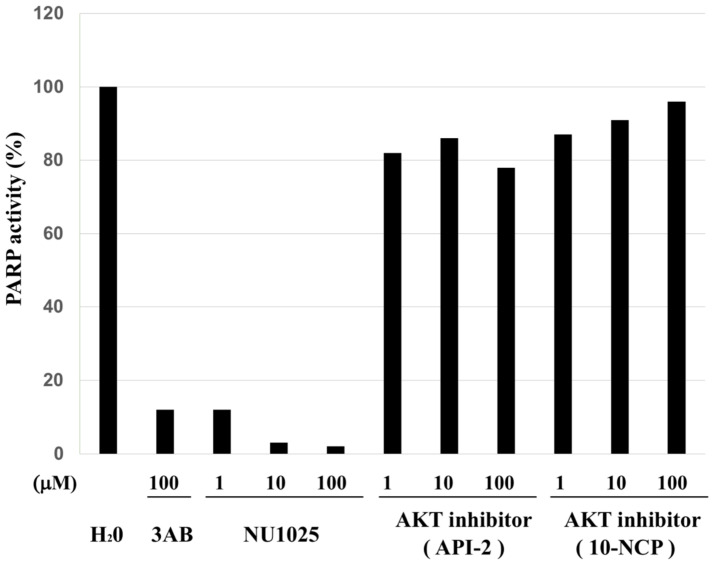
Akt inhibitors did not inhibit PARP1 enzyme activity. Relative PARP1 activity was determined in the presence of defined concentrations of 3AB, API-2 and 10-NCP. H_2_O was used as a negative control.

To exclude the possibility that these AKT inhibitors inhibited PARP activity in vitro, PARP enzyme activity was analyzed. The results showed that these AKT inhibitors did not inhibit PARP1 enzyme activity (Figure 7).

## 3. Discussion

One hundred years ago, Boveri hypothesized that multipolar division will cause abnormal combinations of chromosomes, which leads to the formation of malignant tumors [7,8]. We previously showed that 3AB, a PARP inhibitor that does not induce DNA damage, induced centrosome amplification in CHO-K1 cells [16]. In this study, we further showed that 3AB induced aneuploidy of chromosomes in CHO-K1 cells. In addition, another post-translational modification, Akt phosphorylation, was related to PARylation and was involved in centrosome amplification and aneuploidy of chromosomes. Although PARP inhibitors induce centrosome amplification to mouse embryonic fibroblast cell lines [15], it is not known whether they could induce aneuploidy. From centrosome amplification to aneuploidy, certain additional genetic changes might be required, because CHO-K1 cells are known to have a mutation in the p53 gene [19].

It is interesting to note that PARP inhibitors reduced Akt phosphorylation and AKT inhibitors reduced PARylation in vivo. The precise mechanism of inhibition of cell proliferation by PARP inhibitors and its relationship to PI3K/AKT signaling pathway requires further study. Of note, a combined administration of PI3K inhibitor and PARP inhibitor could be an effective therapy in cancer cells [20,21] as well as in cancer mouse models [22].

It is known that PARP inhibitors will potentiate the therapeutic effects by γ-irradiation or alkylating therapeutic drugs on cancer cells [23]. The potentiating effect of PARP inhibitors might be not only due to the inhibition of DNA repair, but also to the induction of centrosome amplification and aneuploidy to cancer cells. Recently, the combination of aneuploidy and the deletion of certain genes was reported to cause synthetic lethality to cancer cells [24].

Carcinogenesis and cancer therapeutic processes could be two sides of the same coin; thus, we propose centrosome amplification and aneuploidy as another indicator for developing new antitumor drugs against cancers which have been demonstrated to be drug-resistant thus far. Further studies using the primary cell culture and other tissues are required to materialize the concept for clinical fields in the near future.

### Highlights

(1) PARP inhibitor induces centrosome amplification and aneuploidy in CHO-K1 cells;
(2) PARP inhibitors inhibit Akt phosphorylation in CHO-K1 cells;
(3) AKT inhibitors decrease the level of polyADP-ribosylation in CHO-K1 cells;
(4) AKT inhibitors induce centrosome amplification and chromosomal aneuploidy in CHO-K1 cells.

## 4. Material and Methods

### 4.1. Cell and Reagents

Chinese hamster CHO-K1 cells were obtained from Riken (Tsukuba, Japan). CHO-K1 cells were maintained in DMEM supplemented with 10% FBS, 100 U/mL penicillin, and 100 μg/mL streptomycin in an atmosphere containing 5% CO_2_ at 37 °C. 3AB was obtained from Tokyo Chemical Industry Co. (Tokyo, Japan). NU1025 [25] was from Calbiochem (Darmstadt, Germany), and AG14361 [23] was a kind gift from Professor Nicola Curtin (Northern Institute for Cancer Research, Newcastle University, Newcastle Upon Tyne, UK). For immunoblot analysis, the primary antibodies were mouse monoclonal antibodies against α-tubulin (Sigma-Aldrich, Missouri, USA) and PARP1 (BD Transduction Laboratories, New Jersey, USA), rabbit polyclonal antibodies against Akt, Akt (Ser 473) and p44/42 MAPK (ERK1/2) (Cell Signaling Technology, Massachusetts, USA), and rabbit monoclonal antibodies against Phospho-to-Phospho, p44/42 (ERK1/2) (Thr 202/Tyr 204) (Cell Signaling Technology), p38 MAPK and phospho-p38 MAPK (Thr 180/Tyr 182) (EPITOMICS, California, USA)). In addition, horseradish peroxidase (HRP)-conjugated anti-mouse IgG (nacalai tesque, Kyoto, Japan), HRP-conjugated anti-rabbit IgG (nacalai tesque) and HRP-conjugated goat polyclonal antibody against rabbit IgG (Santa Cruz laboratory, Texas, USA) were used as secondary antibodies.

### 4.2. Flow Cytometry

Adherent-culture cells that had been trypsinized were centrifuged at 120× *g* for 3 min and resuspended in PBS. The suspension was fixed with ice-cold 100% ethanol to yield a final concentration of 70%. The suspended cells were centrifuged at 120× *g* for 3 min, resuspended in PBS containing 100 μg/mL RNase A, and stained with propidium iodide. Flow cytometry was performed using FACS Calibur.

### 4.3. Centrosome Amplification Judgement

First, cells were fixed using 100% methanol at −20 °C for 5 min and then washed twice with PBS(-). Subsequently, the cells were solubilized with 1% tritonX-100 in PBS for 4 min at room temperature and washed twice with PBS(-). After blocking with 5% FBS in PBS(-) for 30 min at room temperature, the cells were simultaneously reacted with mouse-derived anti γ-tubulin antibody (SIGMA) (1:300) for 60 min at room temperature and washed three times with PBS(-). Next, antibodies against rabbit IgG were reacted with Alexa488-labeled anti-rabbit IgG antibody (Invitrogen, Massachusetts, USA) (1:2500) as a secondary antibody for 60 min at room temperature under light-shielded conditions and then washed three times with PBS(-). To stain the cell nuclei, cells were incubated with 4′,6-diamino-2-phenylindole dihydrochloride (DAPI) (nacalai tesque) (1:1000) for 10 min at room temperature under light-shielded condition and were then washed three times with PBS(-). Finally, the cells were sealed on glass slides (Fluoromount/Diagnostic BioSystems, Pleasanton, CA, USA), stored at 4 °C under light-shielded conditions overnight, and then observed using a fluorescence microscope (ZEISS FLV1000). The number of spots of γ-tubulin per cell was measured. Cells with more than 2 centrosomes were defined to have centrosome amplification. Each value represents the mean ± SD of 3 independent experiments.

### 4.4. BrdU Labeling Index

The analysis was performed according to the protocol of 5′-Bromo-2′-deoxy-uridine Labeling and Detection kit I (Roche, Switzerland). Briefly, cells were incubated with BrdU for 12 h, washed and fixed with 70% ethanol at −20 °C for 20 min. The cells were then incubated with anti-BrdU antibody at 37 °C for 30 min, washed and incubated with fluorescein-conjugated anti-mouse Ig antibody at 37 °C for 30 min. The cells were washed, sealed and examined under a fluorescence microscope.

### 4.5. Metaphase Spread of Chromosomes

Cells were incubated in the presence of colcemid (0.5 µg/mL) for 6 h to enrich mitotic cells. The medium containing floating mitotic cells was saved. The remaining cells were trypsinized and pelleted together with the saved medium by centrifugation. The cell pellet was then gently resuspended in a hypotonic solution (65 mM KCl) and allowed to stand for 20 min at 37 °C. Next, the hypotonic solution was removed, after which a methanol–acetic acid fixative was added, and the cells were allowed to stand for 3 min. The old fixative was later discarded, and then fresh fixative was added. This procedure was repeated twice. A few drops of the suspension on coverslips were subjected to Giemsa staining and then examined under a light microscope. The frequency of mitotic cells per culture condition was determined by tallying the number of mitotic cells out of a population of 1000 consecutive cells, ignoring broken cells, clumped cells and cellular debris. The mitotic index was determined by multiplying the ratio of mitotic cells to total cells by 100%.

### 4.6. Western Blot Analysis

Cells were lysed using a lysis buffer (20 mM Tris-HCl (pH 7.5), 0.25% sodium deoxycholate, 0.025% sodium dodecyl sulfate (SDS), 150 mM NaCl, 5 mM EDTA, 1 mM NaF, 1 mM NaVO_4_ and 1% NP40), which contained protease inhibitors (EDTA-free protease inhibitor cocktail; Roche Diagnostics). The resulting cell lysates were incubated on ice for 30 min and then centrifuged at 20,000× *g* for 15 min at 4 °C. The proteins present in the supernatant were then denatured in a sample buffer (62.5 mM Tris-HCl (pH 6.8), 10% (*v/v*) glycerol, 2% SDS, 5% 2-mercaptoethanol and 0.001% bromophenol blue), boiled for 5 min, separated using SDS-PAGE and transferred onto an Immobilon P-membrane (Millipore, Massachusetts, USA). The membrane was incubated overnight with primary antibody at 4 °C and then with HRP-conjugated secondary antibody in PBS containing 5% skim milk and 0.05% (*v/v*) Tween 20 for 60 min at room temperature. Next, the membrane was washed with 0.05% Tween 20 in PBS. Immunopositive bands were visualized with enhanced chemiluminescence using LAS3000 imaging systems (Fujifilm, Tokyo, Japan). The intensity of the bands was quantified using Image J.

### 4.7. Sample Preparation for ELISA

The procedure is essentially the same as reported elsewhere [26,27]. Briefly, CHO-K1 cells were washed with PBS and then immediately fixed by adding ice-cold 20% TCA. After standing on ice for 10 min, the cells were collected with a plastic policeman, centrifuged at 800× *g* for 20 min at 4 °C, washed with diethyl ether, dissolved in 200 μL of 0.2 N NaOH, sonicated with a micro tip and incubated for 1 h at 37 °C. Each sample was neutralized with 1 M Tris-HCl (pH 8.0) and 2 N HCl to yield pH 7.5–8.0, and incubated with 20 μg/mL DNase I, 20 μg/mL RNase A, and 1 U/mL nuclease P1 in the presence of 5 mM MgCl_2_ overnight at 37 °C. Then, each sample was digested with 0.2 mg/mL proteinase K overnight at 50 °C, boiled for 5 min and centrifuged at 9700× *g* for 10 min at 4 °C. Each supernatant was frozen at −20 °C until ELISA.

### 4.8. ELISA for Poly(ADP-Ribose) Level

ELISA for poly(ADP-ribose) was carried out essentially as described previously [25,26], with some modifications. The microtiter 96-well plate was coated with a monoclonal antibody against poly(ADP-ribose) (10H) [28] at 10 μg/mL in 50 μL coating buffer (15 mM Na_2_CO_3_ and 37 mM NaHCO_3_) overnight at 4 °C. The wells were blocked with 100 μL of 2% non-fat dry milk in 0.1% (*w/v*) Tween 20 in PBS (0.1% T-PBS) for 1 h at 25 °C and washed three times with 200 μL of 0.05% T-PBS. Samples of purified poly(ADP-ribose) standards were diluted with a buffer (0.1 M mannitol, 0.1 M NaCl and 0.1 M Tris-HCl, pH 8.0), and a 50 μL sample or standard solution containing 0, 2.5, 5, 10, 25, 50 or 75 pg poly(ADP-ribose) was added to each well with subsequent incubation for 2 h at 25 °C. The wells were washed three times with 200 μL of 0.05% T-PBS. A 50 μL volume of rabbit polyclonal anti-poly(ADP-ribose) IgG [29], diluted to 10 μg/mL with 2% non-fat dry milk in 0.1% T-PBS, was added to each well and incubated for 2 h at 25 °C. Wells were washed three times with 200 μL of 0.05% T-PBS. A 50 μL volume of HRP-conjugated goat polyclonal anti-rabbit IgG, diluted to 0.4 μg/mL, was added to each well and incubated for 2 h at 25 °C. The wells were washed five times with 200 μL of 0.05% T-PBS, 100 μL of *o*-phenylenediamine (0.5 mg/mL in 0.05% hydrogen peroxide solution) was added, and they were incubated for 30 min at 25 °C. The reaction was terminated by adding 25 μL of 4 N H_2_SO_4_. Then, the absorbance was measured at 450 nm. Parallel cultures were used for cell counts, and the poly(ADP-ribose) abundance was represented as per 10^6^ cells. Standard poly(ADP-ribose) was prepared as described [30].

### 4.9. PARP Enzyme Activity Assay

Each reaction mixture of 100 μL contained 50 mM Tris-HCl, pH 8.0, 10 mM MgCl_2_, 1 mM DTT, 100 μg/mL BSA, 0.1 mM [^32^P]NAD^+^ (0.2 μCi/μmol), 20 μg/mL of sonicated salmon sperm DNA, 20 μg/mL of calf thymus histone and 3 mU of human PARP1 (Trevigen, PARP-HSA) in the presence or absence of indicated amounts of AKT inhibitors. The reaction was incubated for 10 min at 25 °C and terminated by the addition of 10% TCA. The acid-insoluble reaction products were collected on glass fiber filter discs and washed with 10% TCA, ethanol and ether. The radioactivity was assessed with a scintillation counter.

### 4.10. Statistics

The data are presented as the mean ± standard deviation (SD), and the statistical significance of the difference between subjects was evaluated with two-tailed Student’s *t*-tests. The significance of aneuploidy was analyzed using Fisher’s exact tests.

## Supplementary Materials

The following supporting information can be downloaded at: https://www.mdpi.com/article/10.3390/ijms23073484/s1.
