# Supplementary figures and images for: PARP Inhibitor Decreases Akt Phosphorylation and Induces Centrosome Amplification and Chromosomal Aneuploidy in CHO-K1 Cells
Source: Int J Mol Sci. 2022 Mar 23;23(7):3484. doi: 10.3390/ijms23073484 (PMC8998298; doi:10.3390/ijms23073484)

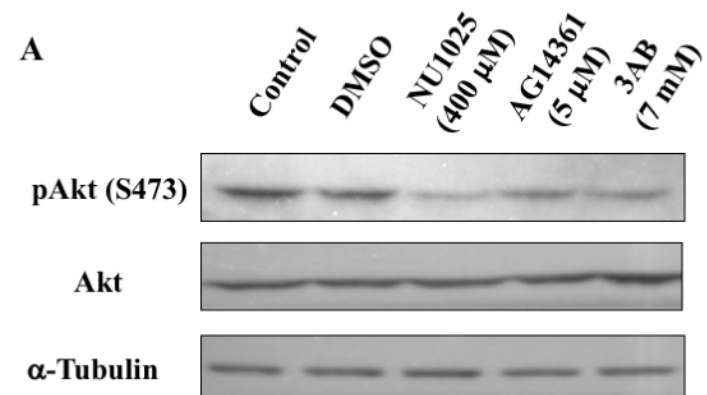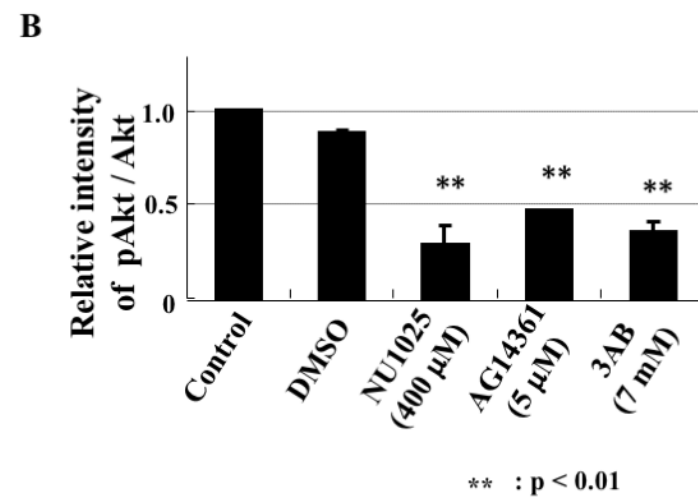

Supplement: Supplementary file 1 [file ijms-23-03484-s001.zip › ijms-1612919-Supplementary Figure S1.pdf]
